# PD03 Exploring Expenditure On State Subsidized Medicines in Ireland Between 2018 And 2022: Special Focus On Cancer Drug Expenditure

**DOI:** 10.1017/S0266462324002733

**Published:** 2025-01-07

**Authors:** Caroline Walsh, Lea Trela-Larsen, Roisin Adams

## Abstract

**Introduction:**

The Department of Health in Ireland published a review of expenditure on state subsidized medicines in August 2021. Detailed analysis indicated exponential growth of expenditure on cancer drugs administered in the community. However, expenditure by drug group across all state subsidized schemes, including hospitals, was not explored.

**Methods:**

Using national reimbursement claims data, total medicines expenditure on community drug schemes (CDS) was analyzed annually for the years 2018 to 2022. The total drug expenditure stratified by anatomical therapeutic class (ATC) code was calculated. Expenditure on cancer drugs (ATC code L) between 2018 and 2022, including hospital data, was further explored. Cancer drugs with the highest expenditure were identified, and trends in their expenditure were analyzed. Dates of European regulatory approval, completion of health-technology assessments by the National Centre for Pharmacoeconomics in Ireland, and reimbursement by the Health Service Executive for the identified cancer drugs were collected.

**Results:**

The total expenditure on drugs on CDS rose from EUR1.74 billion (EUR1.61 billion excluding value-added tax [VAT]) in 2018 to EUR2.2 billion (EUR2.03 billion excluding VAT) in 2022. Expenditure on antineoplastic and immunomodulatory agents (ATC code L) rose from 34 percent in 2018 to 38 percent in 2022. The cancer drugs with the highest cumulative expenditure on hospital schemes were pembrolizumab (EUR98.41 million excluding VAT), nivolumab (EUR75.15 million), daratumumab (EUR54.05 million), and trastuzumab (EUR41.48 million). All the aforementioned drugs demonstrated year-on-year increases in annual expenditure apart from nivolumab. Expenditure on pembrolizumab increased by 48 percent in 2022, compared with 2021.

**Conclusions:**

Expenditure on state subsidized medicines is increasing annually, although confidential discounts may reduce budget impact. Increasing expenditure may be attributed to the expansion of existing indications and increasing patient volume. Expenditure on monoclonal antibodies is substantially larger than expenditure on other drugs. Pembrolizumab was reimbursed for six additional indications in 2021, contributing to the sharp increase in expenditure between 2021 and 2022.

